# Supplementary figures and images for: Self-selected interval judgments compared to point judgments: A weight judgment experiment in the presence of the size-weight illusion
Source: PLoS One. 2022 Mar 16;17(3):e0264830. doi: 10.1371/journal.pone.0264830 (PMC8926213; doi:10.1371/journal.pone.0264830)

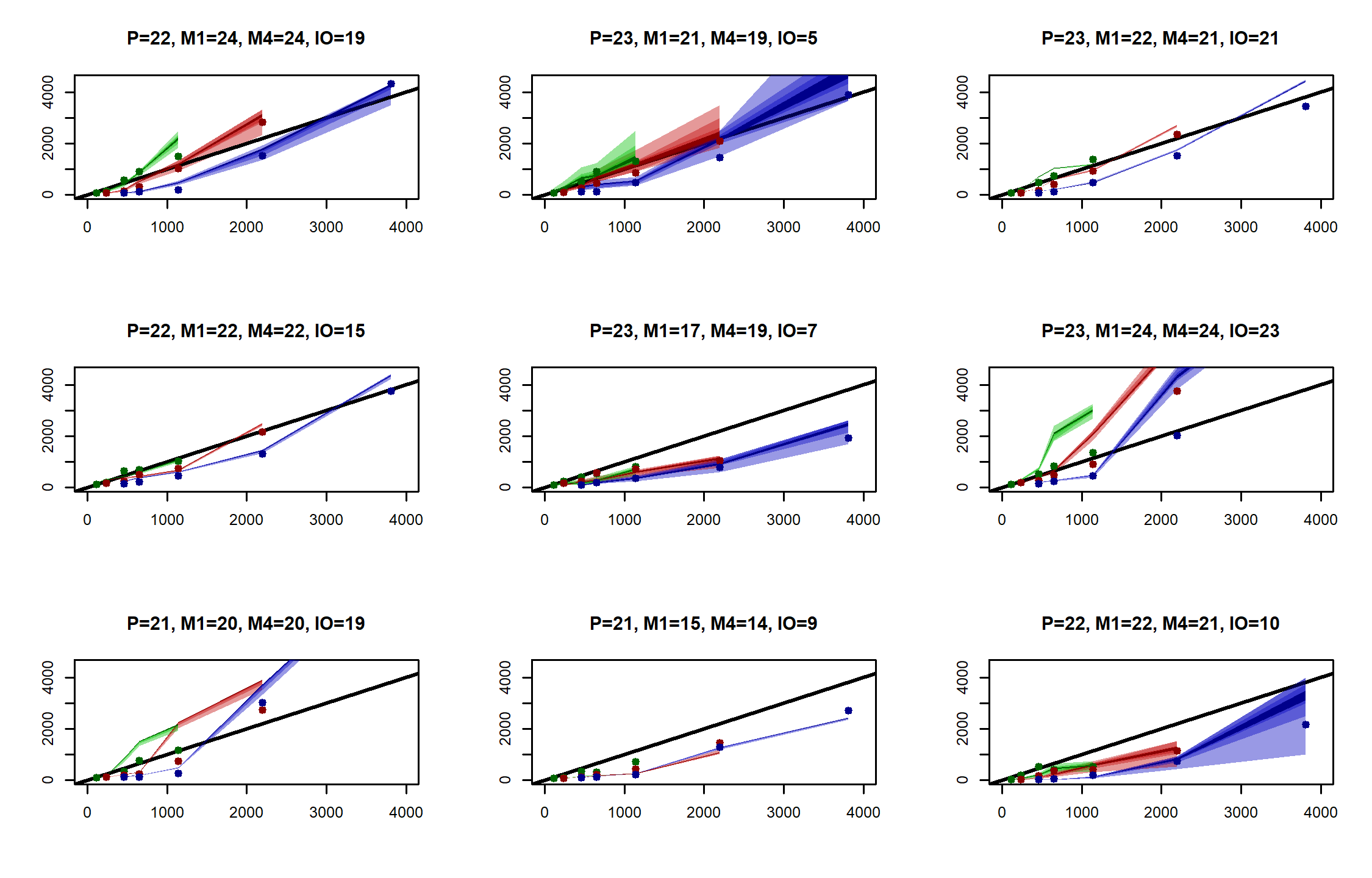

Supplement: S1 Fig — The figure is conceptually the same as Fig 2, which introduces the result section. Points indicate point judgments, intervals indicate the range of the upper and lower interval limits and the color darkens for each consecutive split (i.e. the darkest middle field indicates the upper and lower limits after the final interval split). True weights are described on the x-axis and judgments on the y-axis, thus, the diagonal line indicates true judgments. To these individual plots we have, in the title, added the number of size-weight illusion cases for Point (P), Mid1 (M1), Mid4 (M4) and no interval overlap (IO). (TIFF) [file pone.0264830.s001.tiff]

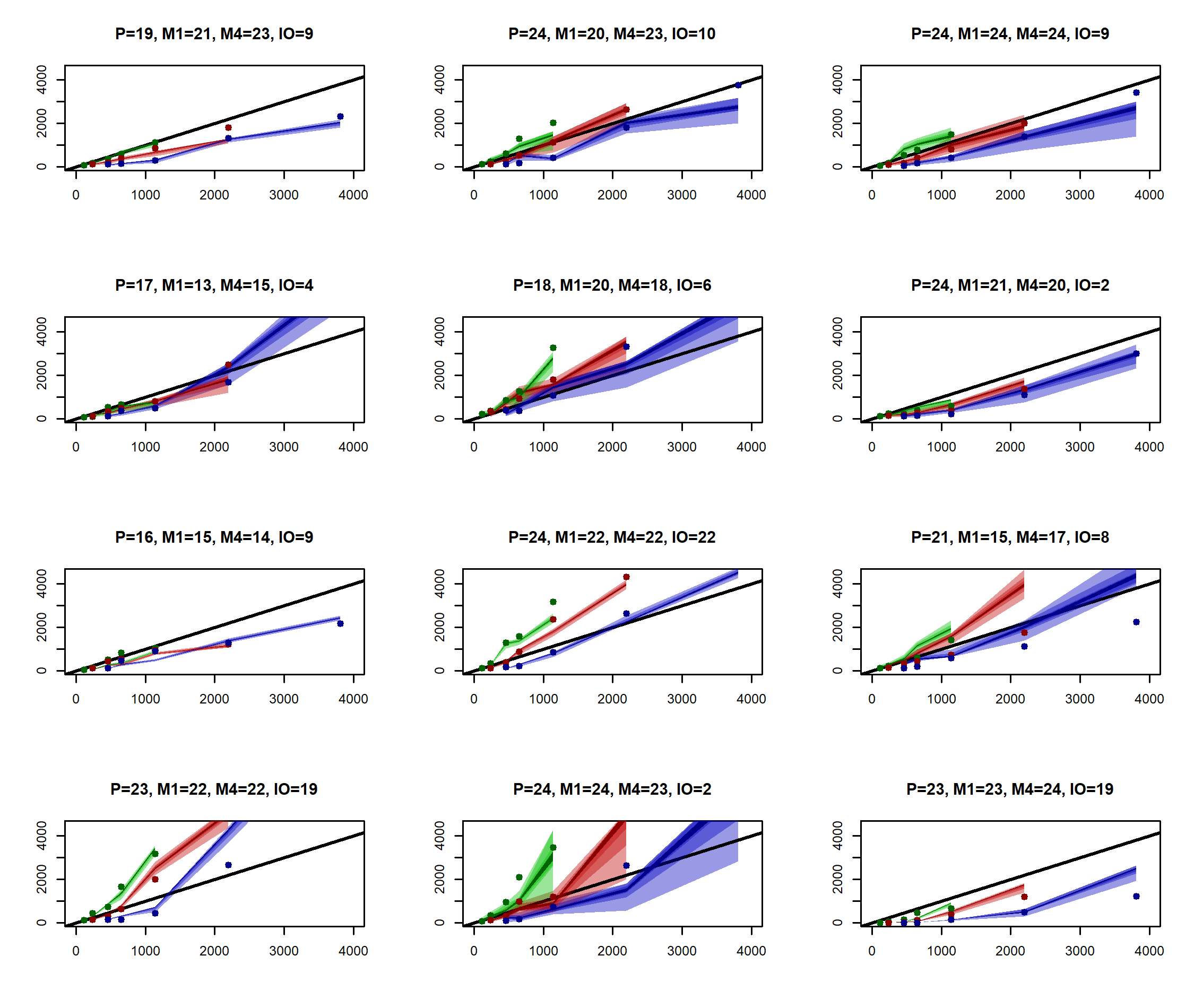

Supplement: S2 Fig — (TIFF) [file pone.0264830.s002.tiff]

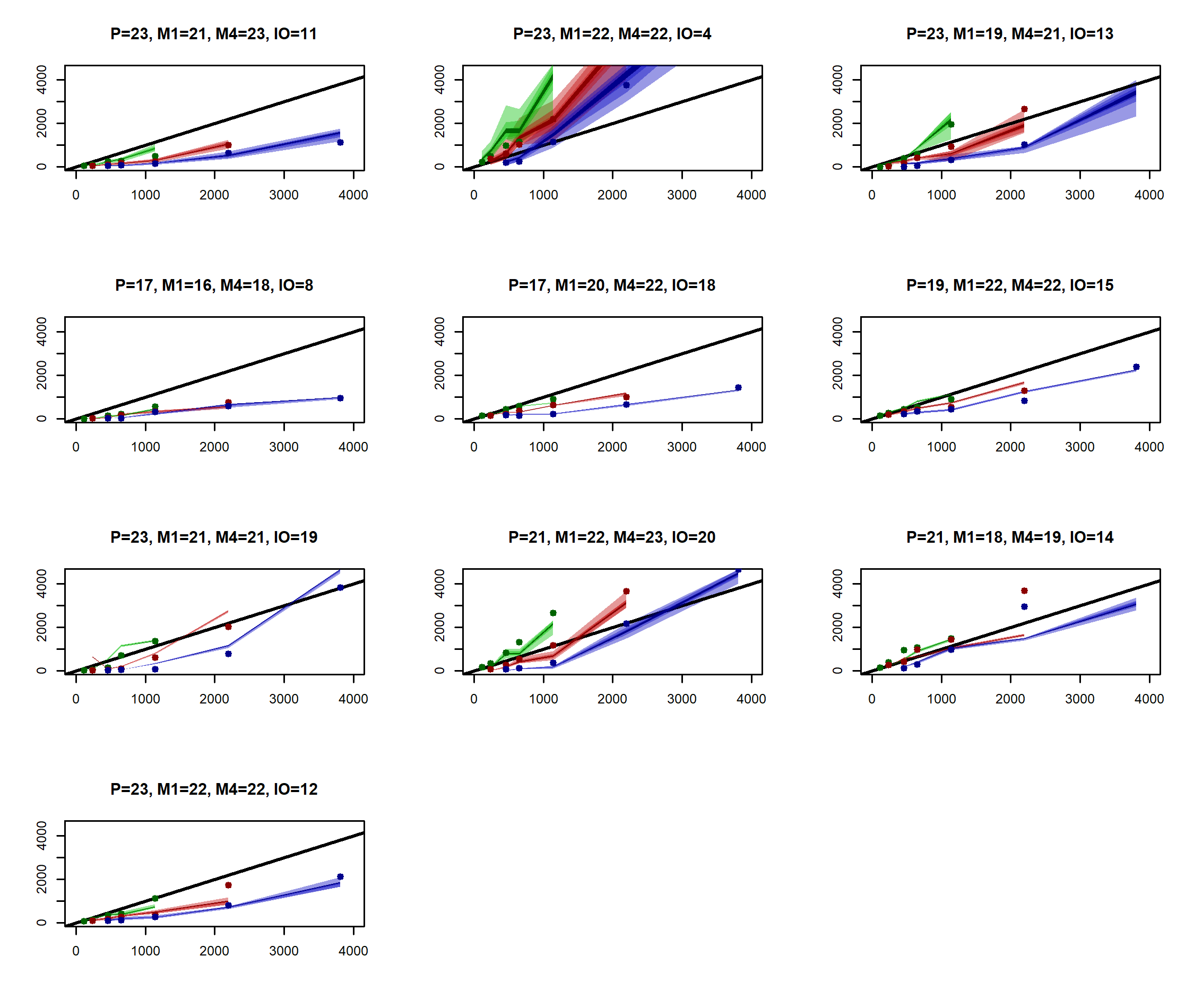

Supplement: S3 Fig — (TIFF) [file pone.0264830.s003.tiff]

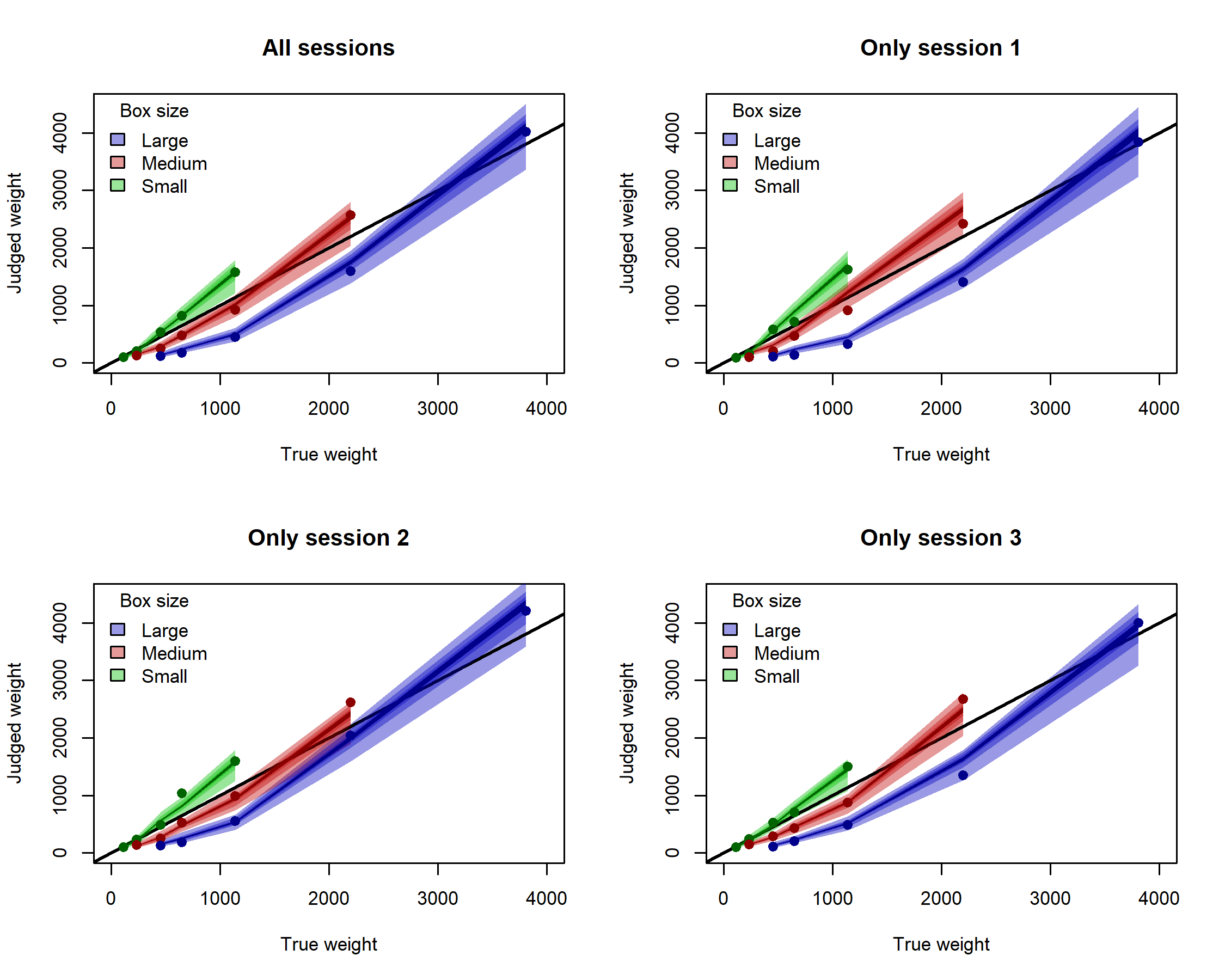

Supplement: S4 Fig — (TIFF) [file pone.0264830.s004.tiff]

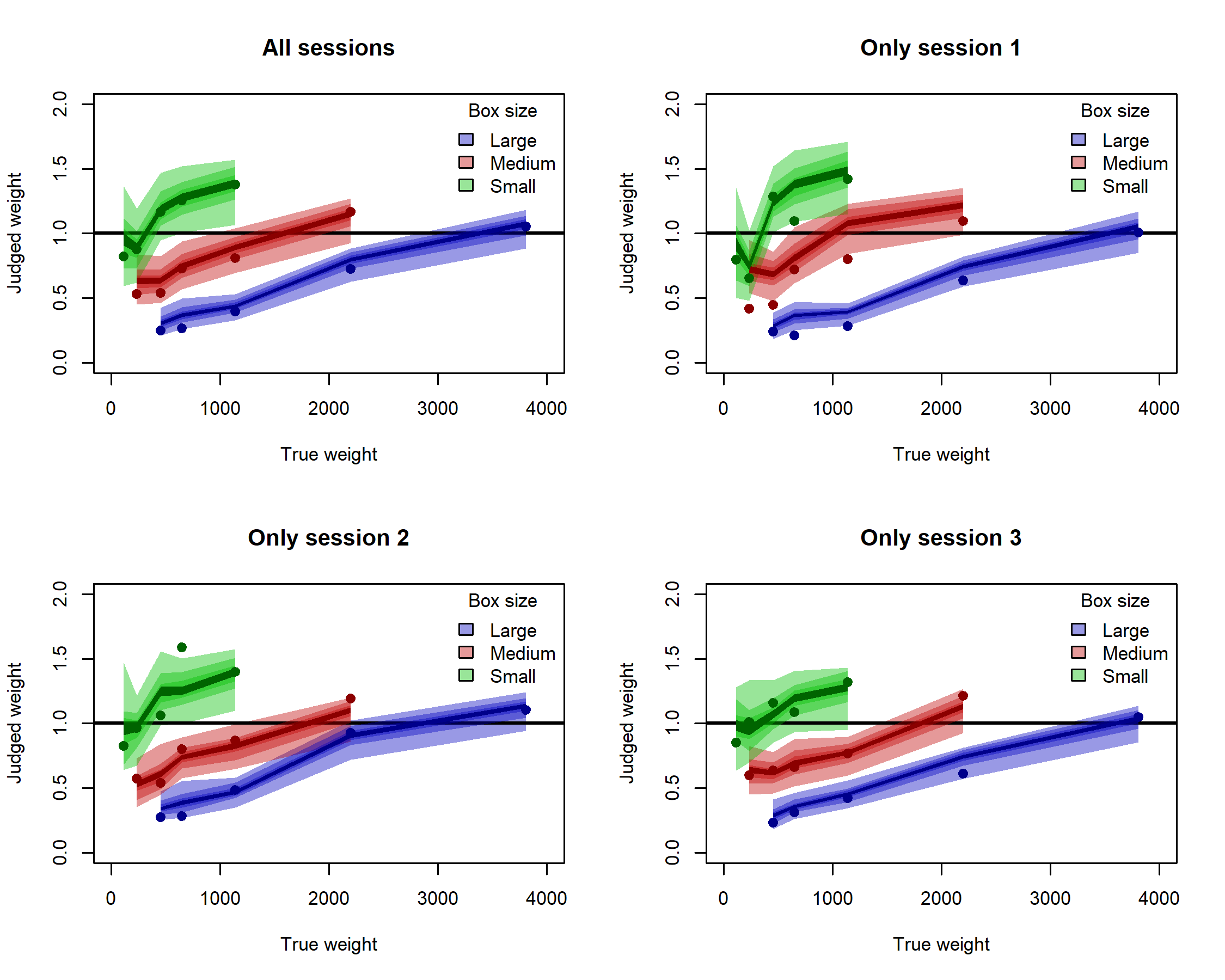

Supplement: S5 Fig — (TIFF) [file pone.0264830.s005.tiff]

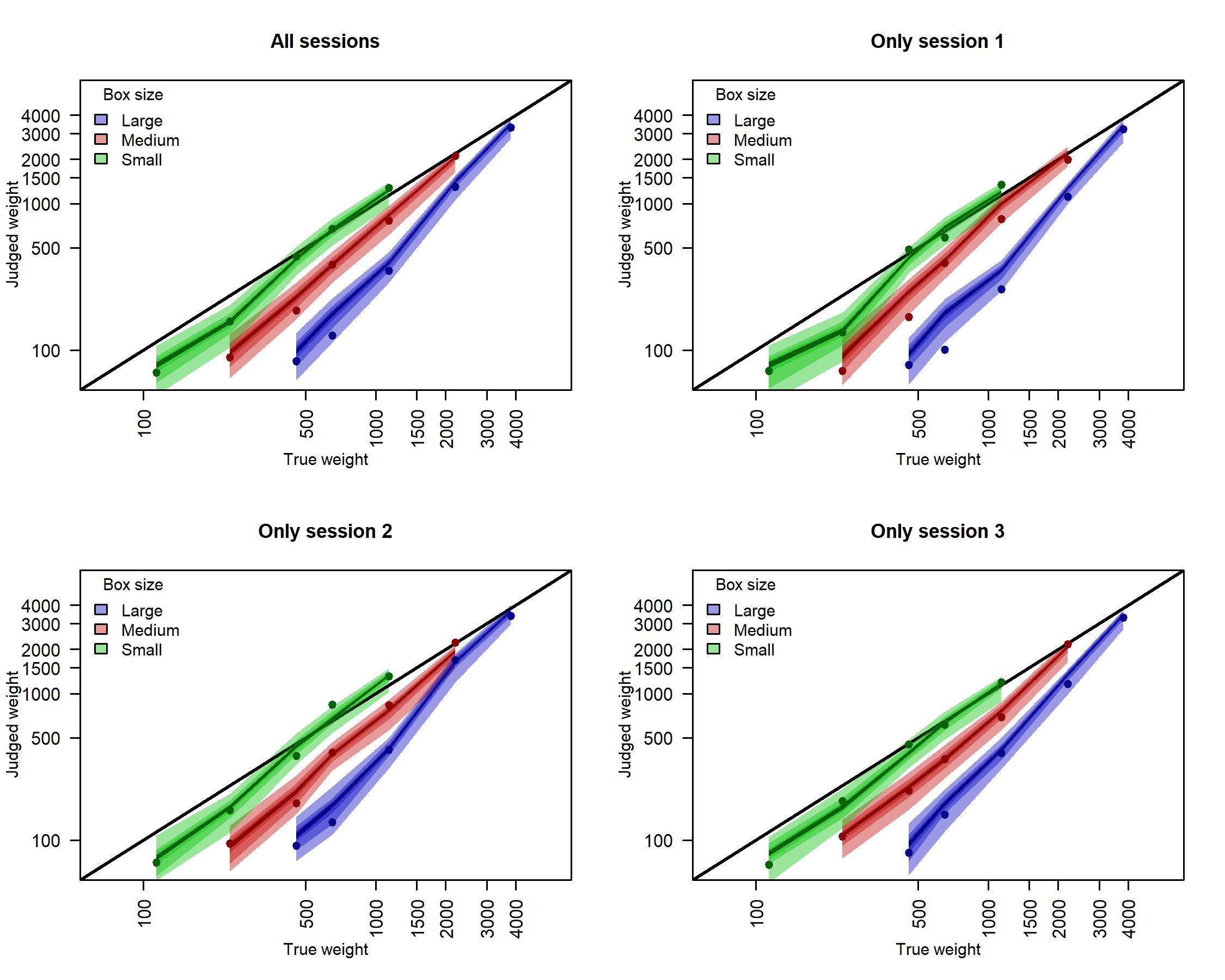

Supplement: S6 Fig — (TIFF) [file pone.0264830.s006.tiff]

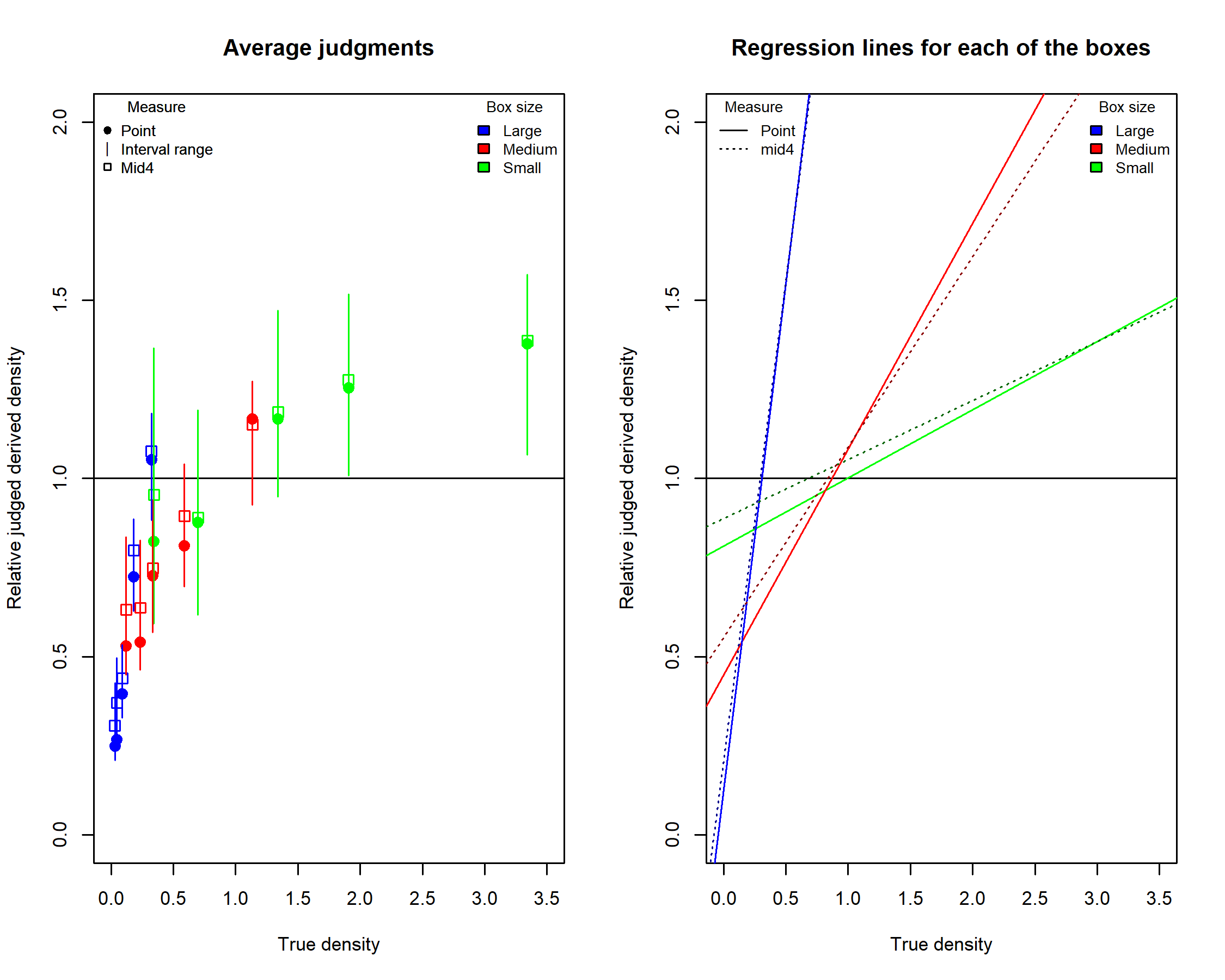

Supplement: S7 Fig — To illustrate how the judgments relate to density depending on box size we first computed the density derived from judgments by dividing weight judgments by the true volume of the judged box. We then calculated the derived judged density relative to the true density. This was done to be able to visualize the full range of judgments in a single plot. Furthermore, with this calculation, a value of 1.0 means that the derived judged density is the same as the true density. The left panel illustrates average judgments for point judgments (solid dots) and mid4 (empty square), along with the average judged upper and lower interval limits (vertical lines showing the range of the interval). The right panel illustrates linear regression lines fitted to the same data used for the left pane. The solid lines are fitted to point judgments and the dotted lines are fitted to the mid4 judgments. The figure shows that the expected density for the different boxes are not the same because the regression lines intersect 1.0 on the y-axis at different points for each of the box sizes, large (blue), medium (red) and small (green). The average expected density for each box size is indicated by the value on the x-axis where the regression line goes through the horizontal line at 1.0 on the y-axis. (TIFF) [file pone.0264830.s007.tiff]
