# Supplementary material for: Self-selected interval judgments compared to point judgments: A weight judgment experiment in the presence of the size-weight illusion
Source: PLoS One. 2022 Mar 16;17(3):e0264830. doi: 10.1371/journal.pone.0264830 (PMC8926213; doi:10.1371/journal.pone.0264830)
Supplement: S1 Table — True weight of boxes and judgment estimates were log transformed for this analysis. Main effects of true box weight, the type of judgment estimate (point vs mid4), and size (small, medium, large). First-order interaction effects between true weights, type of judgment estimate, and size. Random effects for true weight, type of judgement estimate, and size. (DOCX) [file pone.0264830.s008.docx]

**S8 Table. Model estimates: mixed effect model illustrated in Fig 3. True weight of boxes and judgment estimates were log transformed for this analysis.** ***Main effects* of true box weight, the type of judgment estimate (point vs mid4), and size (small, medium, large). *First-order interaction effects* between true weights, type of judgment estimate, and size. *Random effects* for true weight, type of judgement estimate, and size.**

| ***Fixed effects*** | Value | Std.Error | DF | t-value | p-value |
| --- | --- | --- | --- | --- | --- |
| Intercept | -5.88 | 0.51 | 2750 | -11.60 | 0.00 |
| Weight | 1.70 | 0.06 | 2750 | 26.94 | 0.00 |
| Size medium | 2.96 | 0.19 | 2750 | 15.50 | 0.00 |
| Size small | 4.36 | 0.20 | 2750 | 21.90 | 0.00 |
| Estimate point | -0.62 | 0.16 | 2750 | -3.84 | 0.00 |
| Weight : Size medium | -0.33 | 0.03 | 2750 | -12.48 | 0.00 |
| Weight : Size small | -0.47 | 0.03 | 2750 | -18.15 | 0.00 |
| Weight : Estimate point | 0.07 | 0.02 | 2750 | 3.06 | 0.00 |
| Size medium : Estimate point | 0.12 | 0.04 | 2750 | 2.75 | 0.01 |
| Size small : Estimate point | 0.22 | 0.05 | 2750 | 4.62 | 0.00 |
|  |  |  |  |  |  |
| ***Random effects*** | StdDev |  | Corr |  |  |
| Intercept | 2.69 | Intercept | Weight | Point judgments | Size medium |
| Weight | 0.33 | -0.98 |  |  |  |
| Estimate point | 0.25 | -0.16 | 0.07 |  |  |
| Size medium | 0.28 | -0.85 | 0.88 | -0.06 |  |
| Size small | 0.54 | -0.87 | 0.88 | 0.02 | 1.00 |
| Residual | 0.44 |  |  |  |  |

Weight = the log of the true weight of a box. Dummy variables, estimate point = 1 for point judgments and 0 otherwise, size medium = 1 for medium boxes and 0 otherwise, and size small = 1 for small boxes and 0 otherwise.
