# Supplementary material for: Self-selected interval judgments compared to point judgments: A weight judgment experiment in the presence of the size-weight illusion
Source: PLoS One. 2022 Mar 16;17(3):e0264830. doi: 10.1371/journal.pone.0264830 (PMC8926213; doi:10.1371/journal.pone.0264830)
Supplement: Attachment — Submitted filename: Response to Reviwer 1.docx [file pone.0264830.s010.docx]

Dear Dr. Howe

We have revised our manuscript in response to the issues raised by reviwer 1. Please, find below our responses. We have numbered the questions 1 – 9 and marked our responses wit **R** and the corresponding number. Reviewer issues are in italics and responses in normal text.

**Reviewer #1:** I think this is an improved, more substantive version of this paper and I appreciate the authors’ work on the manuscript. However, the authors make some claims based on results/data that they have not tested statistically. This is problematic. There are also some other more minor issues that I believe still need to be addressed before this work is publishable.

**Major issues:**

***1.*** *Line 405 states that there was an ANOVA (actually 2) but then the results are not reported. This is confusing and I still don’t understand why, even they talk about a priori hypotheses etc. In their study aims paragraph in the introduction they state “We also wanted to compare the strength of the illusion across the two different judgment methods”. Then, on line 421 they say “We interpret this as the SWI-reducing effect of the interval method…” as well as similar claims/interpretations in the Discussion. The authors should not claim an effect of a manipulation without having inferential statistical proof of the effect. This needs to be addressed in some way. Similarly, the figure 4 and 5 captions also refer to “interaction plots” which I think is misleading given that they haven’t tested an interaction.*

**R1.** We agree that the section was confusing, and we have rewritten it so that it is clearer that it was specifically the peak that we had no prior hypothesis. We have also added ANOVA-analyses of the main effect of measure and the interaction effect with weight causing the peak for point measures. [lines 406 – 437]

**2.** *The same issue remains regarding variability (ie SD) in judgements on line 537 onwards. They claim differences that have not been tested statistically.*

**R2.** We have added t-tests for these differences. [lines 541 – 542]

**3.** *Lines 620 onwards – this paragraph doesn’t make sense. Not “thinking the illusion away” is what many previous authors have meant by stating it is “cognitively impenetrable”. This is also not what is meant in the literature base by the bottom up vs top down issue. I think the authors needs to research these issues more carefully if they want to include discussion about them.*

**R3.** The intended meaning of this paragraph does not add anything of importance and we have therefore removed it.

**4.** *Also, one of the primary “bottom-up” explanations is related to density perception, which the authors have now referred to in a supplementary figure following my request, but this is still not mentioned in the main manuscript. I still think this is problematic for the reasons I stated in my original review, including that it is reported as an (important) stimulus characteristic. I believe at least a brief discussion of the potential contribution of density to their results and a statement pointing the reader to the supplementary figure is warranted.*

**R4.** We have clarified in the stimuli description that density was used to center the entire stimulus set around a parameter and that it was not intended as a main research topic for this study [lines 175 – 178]. We acknowledge that density matters for SWI studies and it is now discussed briefly along with directions to the figure [lines 612 – 619].

**Minor issues:**

***5.*** *Line 33 “the smaller of two identical (w.r.t shape and size) objects” does not make sense ie there can’t be a smaller of two objects with identical size. Maybe the authors mean weight instead of size?*

**R5.** Thank you for pointing out this clearly confusing sentence, it has been rewritten.

***6.*** *Line 131 onwards: “Furthermore, focusing on weight, rather than size, has been found to increase the illusion (19). This suggests that asking for the true weight should increase the illusion, because it leads the participants to focus on weight. Hence, differences between judgment methods should be easier to find with a strong illusion when the true weight is judged, instead of subjective heaviness being judged.”
I do not agree with the statement starting with “this suggests…”. I think focusing on true weight instead of heaviness is very different from focusing on weight instead of size. My intuition is that the former would decrease the illusion if anything. I’m not suggesting the authors change their prediction on this issue to accord with mine necessarily but I think whatever they predict on the issue needs to be better substantiated than it is currently. Otherwise I’d say it’s best to remain agnostic on the issue if they cannot.*

**R6.** We agree that the reasoning does not hold when assuming that judgments of true weight should increase the illusion more than subjective judgments of heaviness do. The text has been revised accordingly [lines 132 - 134].

**7.** Typo line 168 – “where” instead of “were”. Same on line 197 “boxes where filled with cotton”.

**R7.** We have corrected this, thank you for noticing.

**8.** I think Fig1 was missing.

**R8.** We have checked that the figure should now be included.

**9.** Fig5 – not sure if it is a problem with the figure or just how it uploaded on the system but the line for mid4 is not visible.

**R9.** We have now checked the figures with the Preflight Analysis and Conversion Engine (PACE) as requested by PLOS ONE where no problems are reported and the lines are visible.
